# Supplementary material for: Low-level mosaic variants causing the pancreatic disease congenital hyperinsulinism can be detected from blood DNA
Source: eBioMedicine. 2026 May 25;128:106308. doi: 10.1016/j.ebiom.2026.106308 (PMC13226933; doi:10.1016/j.ebiom.2026.106308)
Supplement: Collab authors [file mmc1.docx]

**The International Congenital Hyperinsulinism Consortium**

Teoman Akçay^1^, Doha Sager Alhomaidah^2^, Nisha Bhavani^3^, Bianca Fiorella Miranda Cabrera^4^, Korcan Demir^5^, Ghaisana Fadiana^6^, Ghadir Elias-Assad^7,8^, Eiroa Hernan^10^, Rajesh Joshi^11^, Manjiri Karlekar^12^, Natalya Karp^13,14^, Vjosa Mulliqi Kotori^15^, Veronica Mericq^16^, Chirantap Oza^17^, Vaman Khadilkar^17^, VP Praveen^3^, Birgit Rami-Merhar^18^, Sumudu Nimali Seneviratne^19^, Zeynep Şıklar^20^, Yardena Tenenbaum Rakover^21^

**Affiliations**

1. Department of Pediatrics, Istanbul Health and Technology University (ISTUN) Medical Faculty, Istanbul, Türkiye.
2. Pediatric Endocrinology, Al Farwaniyah Hospital, Kuwait
3. Department of Endocrinology, Amrita Institute of Medical Sciences, Kochi, India
4. Pediatric Endocrinology Unit, Hospital Nacional Edgardo Rebagliati Martins, Lima, Peru
5. Department of Paediatric Endocrinology, Dokuz Eylül University, Izmir, Türkiye.
6. Cipto Mangunkusumo National General Hospital, Jakarta, Indonesia
7. Pediatric Endocrine Unit, Saint Vincent de Paul Hospital, Nazareth, Israel.
8. Azrieli Faculty of Medicine, Bar-Ilan University, Israel.
9. Department of Endocrinology, The Hospital for Sick Children, Toronto, Canada
10. Hospital Juan P. Garrahan, Ciudad de Buenos Aires, Argentina
11. B J wadia Hospital for Children, Acharya Donde Marg, Parel, Mumbai, India
12. Department of Endocrinology and Metabolism, Seth G. S. Medical College and KEM Hospital, Mumbai, India.
13. Western University, Department of Pediatrics, Division of Medical Genetics, London, Canada.
14. London Health Sciences Centre, Medical Genetics Program, Canada.
15. UBT College, Prishtina, Kosova
16. Institute of Maternal and Child Research, University of Chile, Santiago, Chile.
17. Jehangir Hospital, Pune, India
18. Dept. of Pediatric and Adolescent Medicine, Comprehensive Center for Pediatrics, Medical University of Vienna, Austria
19. Faculty of Medicine, University of Colombo, Colombo, Sri Lanka
20. Ankara University School of Medicine, Department of Pediatric Endocrinology, Ankara, Türkiye.
21. Consulting Center, Clalit Health Services, Afula, Israel

| First Names | Surnames |
| --- | --- |
| Teoman | Akçay |
| Doha Sager | Alhomaidah |
| Nisha | Bhavani |
| Bianca Fiorella | Miranda Cabrera |
| Korcan | Demir |
| Ghaisana | Fadiana |
| Ghadir | Elias-Assad |
| Eiroa | Hernan |
| Rajesh | Joshi |
| Manjiri | Karlekar |
| Natalya | Karp |
| Vjosa | Mulliqi Kotori |
| Veronica | Mericq |
| Chirantap | Oza |
| Vaman | Khadilkar |
| VP | Praveen |
| Birgit | Rami-Merhar |
| Sumudu Nimali | Seneviratne |
| Zeynep | Şıklar |
| Yardena | Tenenbaum Rakover |
