# Supplementary material for: Low-level mosaic variants causing the pancreatic disease congenital hyperinsulinism can be detected from blood DNA
Source: eBioMedicine. 2026 May 25;128:106308. doi: 10.1016/j.ebiom.2026.106308 (PMC13226933; doi:10.1016/j.ebiom.2026.106308)
Supplement: Supplementary material [file mmc2.docx]

**Supplementary Information**

**Supplementary Table 1:** Minimum gene list in which disease-causing homozygous, heterozygous and high-level mosaic variants had been excluded in individuals with congenital hyperinsulinism and neonatal diabetes.

| **Disease** | **Genes screened** |
| --- | --- |
| Congenital Hyperinsulinism | *ABCC8, CACNA1D, GCK, GLUD1, HADH, HK1 (non-coding regulatory region), HNF1A, HNF4A, INSR, KCNJ11, PMM2, SLC16A1 (promoter),* and *TRMT10A* |
| Neonatal Diabetes | *KCNJ11*, *ABCC8, INS, EIF2AK3, FOXP3, GATA4, GATA6, GCK, GLIS3, HNF1B, IER3IP1, NEUROD1, NEUROG3, NKX2-2, PDX1, PTF1A, RFX6, SLC2A2, SLC19A2, STAT3, WFS1,*and *ZFP57* |

**Supplementary Table 2:** Mean read depth of targeted next generation sequencing (tNGS) data over the exons of *ABCC8*, *GCK*, *GLUD1*, *KCNJ11,* and *INS*, and the non-coding regulatory region of *HK1* in samples from individuals with genetically undiagnosed congenital hyperinsulinism (CHI, n=1,252) and neonatal diabetes (NDM, n=312).

| **Gene** | **Disease (n samples)** | **Minimum mean read depth for 90% of samples** |
| --- | --- | --- |
| *ABCC8* | CHI (n=1,252) and NDM (n=312) | 400 |
| *GCK* | CHI (n=1,252) | 400 |
| *GLUD1* | CHI (n=1,252) | 413 |
| *HK1* (non-coding 46bp region of interest) | CHI (n=271) | 581 |
| *KCNJ11* | NDM (n=312) | 278 |
| *INS* | NDM (n=312) | 394 |

**Supplementary Table 3:** Curated list of known dominant disease-causing congenital hyperinsulinism variants screened for in this study, where a heterozygous control was available allowing for ddPCR testing. The coding location of coding variants are provided according to the following transcripts *ABCC8*: NM_001287174.1, *GCK*: NM_000162.3, *GLUD1*: NM_005271.4. The genomic locations of the reported *HK1* variants in the CHI-causing region are provided according to GRCh38.

| **Gene** | **Variant** | **Evidence** |
| --- | --- | --- |
| *ABCC8* | Q474R, c.1421A>G | Reported in (1) |
| *ABCC8* | A478D, c.1433C>A | Reported in (1) |
| *ABCC8* | V715M, c.2143G>A | Reported in (2) |
| *ABCC8* | G716D, c.2147G>A | Reported in (3) |
| *ABCC8* | E825K, c.2473G>A | Reported in (3) |
| *ABCC8* | A1153V, c.3458C>T | Reported in (4) |
| *ABCC8* | A1153T, c.3457G>A | Reported in (5) |
| *ABCC8* | R1353H, c.4058G>A | Reported in (6) |
| *ABCC8* | G1384E, c.4151G>A | Reported in (1) |
| *ABCC8* | S1387del, c.4160_4162del | Reported in (7) |
| *ABCC8* | S1387F, c.4160C>T | Reported in (3) |
| *ABCC8* | L1390R, c.4169T>G | Reported in (8) |
| *ABCC8* | L1431F, c.4291C>T | Reported in (8) |
| *ABCC8* | Q1459E, c.4375C>G | Reported in (8) |
| *ABCC8* | Q1459H, c.4377G>C | Reported in (1) |
| *ABCC8* | G1478V, c.4433G>T | Reported in (7) |
| *ABCC8* | G1479A, c.4436G>C | Reported in (9) |
| *ABCC8* | G1479R, c.4435G>A | Reported in (8) |
| *ABCC8* | G1485E, c.4454G>A | Reported in (10) |
| *ABCC8* | G1485R, c.4453G>A | Reported in (11) |
| *ABCC8* | G1488R, c.4463A>G | Reported in (4) |
| *ABCC8* | D1506E, c.4518C>A | Reported in (10) |
| *ABCC8* | D1506E, c.4518C>G | Reported in (3) |
| *ABCC8* | D1506H, c.4516G>C | Reported in (12) |
| *ABCC8* | D1506N, c.4516G>A | Reported in (4) |
| *ABCC8* | E1507K, c.4519G>A | Reported in (13) |
| *ABCC8* | A1508P, c.4522G>C | Reported in (8) |
| *ABCC8* | I1512T, c.4535T>C | Reported in (14) |
| *ABCC8* | M1514K, c.4541T>A | Reported in (10) |
| *ABCC8* | A1537V, c.4610C>T | Reported in (8) |
| *ABCC8* | R1539Q, c.4616G>A | Reported in (8) |
| *GCK* | S64Y, c.191C>A | Reported in (15) |
| *GCK* | T65I, c.194C>T | Reported in (15) |
| *GCK* | T65A, c.193A>G | Reported in (16) |
| *GCK* | G68V, c.203G>T | Reported in (17) |
| *GCK* | S69P, c.205T>C | Reported in (18) |
| *GCK* | K90T, c.269A>C | Reported in (19) |
| *GCK* | V91L, c.271G>T | Reported in (18) |
| *GCK* | W99R, c.295T>A | Reported in (15) |
| *GCK* | T103S, c.308C>G | Reported in (20) |
| *GCK* | M197I, c.591G>A | Reported in (21) |
| *GCK* | Y214C, c.641A>G | Reported in (22) |
| *GCK* | V389L, c.1165G>C | Reported in (20) |
| *GCK* | R447L, c.1340G>T | Reported in (23) |
| *GCK* | A454dup, c.1361_1363dup | Reported in (21) |
| *GLUD1* | R274C, c.820C>T | Reported as R221C in (24) |
| *GLUD1* | H315Y, c.943C>T | Reported in (25) |
| *GLUD1* | R318K, c.953G>A | Reported as R265K in (24) |
| *GLUD1* | R322H, c.965G>A | Reported as R269H in (24) |
| *GLUD1* | N463D, c.1387A>G | Reported as N410D in (24) |
| *GLUD1* | N463T, c.1388A>C | Reported as N410T in (24) |
| *GLUD1* | P489L, c.1466C>T | Reported as P436L in (24) |
| *GLUD1* | Q494R, c.1481A>G | Reported as Q411R in (26) |
| *GLUD1* | S498L, c.1493C>T | Reported in (27) |
| *GLUD1* | G499S, c.1495G>A | Reported in (27) |
| *GLUD1* | G499R, c.1495G>C | Reported as G446R in (26) |
| *GLUD1* | G499A, c.1496G>C | Reported as G446A in (28) |
| *GLUD1* | G499V, c.1496G>T | Reported in (29) |
| *GLUD1* | A500T, c.1498G>A | Reported as A447T in (26) |
| *GLUD1* | H507Y, c.1519C>T | Reported in (27) |
| *HK1* | 10:69348869-69348890delins19 | Reported as 10:71108625_71108646delins19 in (30) |
| *HK1* | 10:69348885-69348921delins5 | Reported as 10:71108641_71108677delinsAGTAT in (30) |
| *HK1* | 10:69348886G>T | Reported as 10:71108642G>T in (30) |
| *HK1* | 10:69348889T>C | Reported as 10:71108645T>C in (30) |
| *HK1* | 10:69348891C>G | Reported as 10:71108647C>G in (30) |
| *HK1* | 10:69348891C>T | Reported as 10:71108647C>T in (31) |
| *HK1* | 10:69348891C>A | Reported as 10:71108647C>A in (30) |
| *HK1* | 10:69348892C>A | Reported as 10:71108648C>A in (31) |
| *HK1* | 10:69348892C>G | Reported as 10:71108648C>G in (31) |
| *HK1* | 10:69348892C>T | Reported as 10:71108648C>T in (31) |
| *HK1* | 10:69348892del | Reported as 10:71108648del in (30) |
| *HK1* | 10:69348895_69348912del | Reported as 10:71108651_71108668del in (30) |
| *HK1* | 10:69348904_69348932del | Reported as 10:71108660_71108688del in (30) |
| *HK1* | 10:69348908T>G | Reported as 10:71108664T>G in (30) |
| *HK1* | 10:69348909C>G | Reported as 10:71108665C>G in (31) |
| *HK1* | 10:69348909del | Reported as 10:71108665del in (30) |
| *HK1* | 10:69348928-69348929del | Reported as 10:71108684_71108685del in (30) |
| *HK1* | 10:69348931T>C | Reported as 10:71108687T>C in (30) |
| *HK1* | 10:69348932_69348935del | Reported as 10:71108688_71108691del in (31) |

**Supplementary Table 4:** Curated list of known dominant *ABCC8* and *KCNJ11* gain-of-function variants and dominant *INS* neonatal diabetes variants screened for in this study, where a heterozygous control was available allowing for ddPCR testing. The location of variants are provided according to NM_001185098.2 (*INS*), NM_001287174.1 (*ABCC8*) and NM_000525.3 (*KCNJ11*).

| **Gene** | **Variant** | **Evidence** |
| --- | --- | --- |
| *ABCC8* | I49F, c.145A>T | Reported in (32) |
| *ABCC8* | V86A, c.257T>C | Reported in (33) |
| *ABCC8* | F132L, c.394T>C | Reported in (34) |
| *ABCC8* | F132V, c.394T>G | Reported in (35) |
| *ABCC8* | L135P, c.404T>C | Reported in (36) |
| *ABCC8* | P206L, c.617C>T | Reported in (4) |
| *ABCC8* | E208K, c.622G>A | Reported in (35) |
| *ABCC8* | D209N, c.625G>A | Reported in (37) |
| *ABCC8* | D209E, c.627C>A | Reported in (38) |
| *ABCC8* | Q211K, c.631C>A | Reported in (39) |
| *ABCC8* | D212N, c.634G>A | Reported in (38) |
| *ABCC8* | D212Y, c.634G>T | Reported in (33) |
| *ABCC8* | D212G, c.635A>G | Reported in (4) |
| *ABCC8* | D212E, c.636C>G | Reported in (4) |
| *ABCC8* | V215I, c.643G>A | Reported in (40) |
| *ABCC8* | L225P, c.674T>C | Reported in (35) |
| *ABCC8* | A235V, c.704C>T | Reported in (4) |
| *ABCC8* | R306H, c.917G>A | Reported in (36) |
| *ABCC8* | V324M, c.970G>A | Reported in (38) |
| *ABCC8* | V360A, c.1079T>C | Reported in (4) |
| *ABCC8* | L451P, c.1352T>C | Reported in (38) |
| *ABCC8* | S532G, c.1594A>G | Reported in (41) |
| *ABCC8* | F536S, c.1607T>C | Reported in (4) |
| *ABCC8* | F577L, c.1731T>G | Reported in (4) |
| *ABCC8* | I585T, c.1754T>C | Reported in (42) |
| *ABCC8* | V587D, c.1760T>A | Reported in (4) |
| *ABCC8* | R826W, c.2476C>T | Reported in (36) |
| *ABCC8* | G833D, c.2498G>A | Reported in (43) |
| *ABCC8* | H863Y, c.2587C>T | Reported in (44) |
| *ABCC8* | H1024Y, c.3070C>T | Reported in (45) |
| *ABCC8* | N1123D, c.3367A>G | Reported in (46) |
| *ABCC8* | E1141G, c.3422A>G | Reported in (4) |
| *ABCC8* | A1153G, c.3458C>G | Reported in (4) |
| *ABCC8* | F1182L, c.3546C>A | Reported in (4) |
| *ABCC8* | R1183W, c.3547C>T | Reported in (36) |
| *ABCC8* | R1183Q, c.3548G>A | Reported in (38) |
| *ABCC8* | P1199S, c.3595C>T | Reported in (4) |
| *ABCC8* | P1199Q, c.3596C>A | Reported in (4) |
| *ABCC8* | P1199L, c.3596C>T | Reported in (47) |
| *ABCC8* | G1256S, c.3766G>A | Reported in (48) |
| *ABCC8* | L1295F, c.3883C>T | Reported in (4) |
| *ABCC8* | R1314H, c.3941G>A | Reported in (36) |
| *ABCC8* | R1380C, c.4138C>T | Reported in (38) |
| *ABCC8* | R1380H, c.4139G>A | Reported in (38) |
| *ABCC8* | R1380P, c.4139G>C | Reported in (4) |
| *ABCC8* | R1380L, c.4139G>T | Reported in (36) |
| *ABCC8* | T1381N, c.4142C>A | Reported in (4) |
| *ABCC8* | S1501R, c.4503C>A | Reported in (49) |
| *ABCC8* | E1507G, c.4520A>G | Reported in (50) |
| *ABCC8* | E1507D, c.4521G>T | Reported in (50) |
| *ABCC8* | V1523M, c.4567G>A | Reported in (51) |
| *ABCC8* | V1524M, c.4570G>A | Reported in (52) |
| *ABCC8* | A1537P, c.4609G>C | Reported in (53) |
| *ABCC8* | V1540M, c.4618G>A | Reported in (4) |
| *INS* | ?, c.188-31G>A | Reported in (54) |
| *INS* | A24D, c.71C>A | Reported in (55) |
| *INS* | A24V, c.71C>T | Reported in (56) |
| *INS* | L30Q, c.89T>A | Reported in (57) |
| *INS* | G32S, c.94G>A | Reported in (55) |
| *INS* | G32R, c.94G>C | Reported in (55) |
| *INS* | C43S, c.127T>A | Reported in (58) |
| *INS* | C43G, c.127T>G | Reported in (55) |
| *INS* | F48C, c.143T>G | Reported in (59) |
| *INS* | R89C, c.265C>T | Reported in (55) |
| *INS* | C96Y, c.287G>A | Reported in (55) |
| *INS* | C96S, c.287G>C | Reported in (59) |
| *INS* | C96R, c.286T>C | Reported in (58) |
| *KCNJ11* | L17P, c.50T>C | Reported in (4) |
| *KCNJ11* | Q30_R34del, c.81_95del | Reported in (60) |
| *KCNJ11* | K39R, c.116A>G | Reported in (43) |
| *KCNJ11* | C42R, c.124T>C | Reported in (33) |
| *KCNJ11* | H46Y, c.136C>T | Reported in (61) |
| *KCNJ11* | H46L, c.137A>T | Reported in (62) |
| *KCNJ11* | N48I, c.143A>T | Reported in (63) |
| *KCNJ11* | I49F, c.145A>T | Reported in (4) |
| *KCNJ11* | R50G, c.148C>G | Reported in (46) |
| *KCNJ11* | R50Q, c.149G>A | Reported in (61) |
| *KCNJ11* | R50P, c.149G>C | Reported in (64) |
| *KCNJ11* | E51A, c.152A>C | Reported in (65) |
| *KCNJ11* | E51G, c.152A>G | Reported in (4) |
| *KCNJ11* | Q52R, c.155A>G | Reported in (66) |
| *KCNJ11* | Q52L, c.155A>T | Reported in (67) |
| *KCNJ11* | G53S, c.157G>A | Reported in (68) |
| *KCNJ11* | G53R, c.157G>C | Reported in (68) |
| *KCNJ11* | G53D, c.158G>A | Reported in (61) |
| *KCNJ11* | G53V, c.158G>T | Reported in (69) |
| *KCNJ11* | V59M, c.175G>A | Reported in (66) |
| *KCNJ11* | V59A, c.176T>C | Reported in (47) |
| *KCNJ11* | V59G, c.176T>G | Reported in (66) |
| *KCNJ11* | F60Y, c.179T>A | Reported in (70) |
| *KCNJ11* | V64M, c.190G>A | Reported in (47) |
| *KCNJ11* | W68R, c.202T>C | Reported in (71) |
| *KCNJ11* | W68G, c.202T>G | Reported in (72) |
| *KCNJ11* | V129M, c.385G>A | Reported in (4) |
| *KCNJ11* | A161T, c.481G>A | Reported in (73) |
| *KCNJ11* | L164P, c.491T>C | Reported in (61) |
| *KCNJ11* | C166Y, c.497G>A | Reported in (61) |
| *KCNJ11* | C166F, c.497G>T | Reported in (74) |
| *KCNJ11* | I167L, c.499A>C | Reported in (75) |
| *KCNJ11* | M169T, c.506T>C | Reported in (4) |
| *KCNJ11* | K170T, c.509A>C | Reported in (61) |
| *KCNJ11* | K170R, c.509A>G | Reported in (40) |
| *KCNJ11* | K170N, c.510G>C | Reported in (53) |
| *KCNJ11* | A174G, c.521C>G | Reported in (46) |
| *KCNJ11* | E179K, c.535G>A | Reported in (4) |
| *KCNJ11* | E179A, c.536A>C | Reported in (38) |
| *KCNJ11* | I182V, c.544A>G | Reported in (68) |
| *KCNJ11* | I182T, c.545T>C | Reported in (76) |
| *KCNJ11* | K185Q, c.553A>C | Reported in (77) |
| *KCNJ11* | K185T, c.554A>C | Reported in (73) |
| *KCNJ11* | R201S, c.601C>A | Reported in (78) |
| *KCNJ11* | R201G, c.601C>G | Reported in (47) |
| *KCNJ11* | R201C, c.601C>T | Reported in (66) |
| *KCNJ11* | R201H, c.602G>A | Reported in (66) |
| *KCNJ11* | R201L, c.602G>T | Reported in (79) |
| *KCNJ11* | E227K, c.679G>A | Reported in (80) |
| *KCNJ11* | E229K, c.685G>A | Reported in (38) |
| *KCNJ11* | L233F, c.697C>T | Reported in (81) |
| *KCNJ11* | V252M, c.754G>A | Reported in (65) |
| *KCNJ11* | V252L, c.754G>C | Reported in (82) |
| *KCNJ11* | V252A, c.755T>C | Reported in (83) |
| *KCNJ11* | V252G, c.755T>G | Reported in (65) |
| *KCNJ11* | P254Q, c.761C>A | Reported in (84) |
| *KCNJ11* | I284F, c.850A>T | Reported in (85) |
| *KCNJ11* | E292G, c.875A>G | Reported in (83) |
| *KCNJ11* | T293N, c.878C>A | Reported in (86) |
| *KCNJ11* | I296L, c.886A>C | Reported in (66) |
| *KCNJ11* | E322K, c.964G>A | Reported in (87) |
| *KCNJ11* | V328M, c.982G>A | Reported in (4) |
| *KCNJ11* | Y330C, c.989A>G | Reported in (88) |
| *KCNJ11* | S331P, c.991T>C | Reported in (4) |
| *KCNJ11* | F333L, c.997T>C | Reported in (89) |
| *KCNJ11* | G334S, c.1000G>A | Reported in (4) |
| *KCNJ11* | G334R, c.1000G>C | Reported in (4) |
| *KCNJ11* | G334C, c.1000G>T | Reported in (73) |
| *KCNJ11* | G334D, c.1001G>A | Reported in (90) |
| *KCNJ11* | G334V, c.1001G>T | Reported in (91) |

**Supplementary Table 5**: Known disease-causing dominant variants called by GATK3 mutect2. The coding location of variants are provided according to the following transcripts *ABCC8*: NM_001287174.1, *GCK*: NM_000162.3, *GLUD1*: NM_005271.4. The genomic locations of the *HK1* variants are provided according to GRCh38. Data on the alternative (ALT) and reference (REF) allele counts, along with the variant allele frequencies (VAF) and 95% confidence intervals (CI) are provided for both the tNGS and ddPCR data. Rows which are not highlighted denote variants that were confirmed by ddPCR (true positives), whilst rows highlighted in grey represent false positive calls that were not confirmed by ddPCR. ^‡^Indicates the two variants identified in one individual. ^†^Indicates variants not called by GATK4 mutect2. ^§^Samples with evidence of evidence of low-level contamination. *Low ddPCR VAF likely due to binding of reference allele probe to alternate allele probe. Unable to control out. **One replicate due to failing of column A.

| **Disease** | **Gene** | **Variant** | **tNGS ALT:REF allele count** | **tNGS VAF % (95% CI)** | **ddPCR ALT:REF droplets** | **ddPCR % (95% CI)** |
| --- | --- | --- | --- | --- | --- | --- |
| HI | *ABCC8* | c.4454G>A, p.(Gly1485Glu) | 27:317 | 7.8 (5.2-11.2) | 637:5027 | 11.2 (10.4-12.1) |
| HI | *ABCC8* | c.4151G>A, p.(Gly1384Glu) | 70:940 | 6.9 (5.4-8.7) | 489:8156 | 5.7 (5.2-6.2) |
| HI | *GCK* | c.641A>G, p.(Tyr214Cys) | 28:401 | 6.5 (4.4-9.3) | 476:7890 | 5.7 (5.2-6.2) |
| HI | *HK1* | Chr10:g.69348932_69348935del | 31:471 | 6.2 (4.2-8.7) | 292:4936 | 5.6 (5.0-6.2) |
| HI | *GCK* | c.1361_1363dup, p.(Ala454dup) | 59:932 | 6.0 (4.6-7.6) | 352:5917 | 5.6 (5.1-6.2) |
| HI | *GLUD1* | c.965G>A, p.(Arg322His) | 80:1272 | 5.9 (4.7-7.3) | 340:15993 | 2.1 (1.9-2.3)* |
| HI | *GLUD1* | c.1493C>T, p.(Ser498Leu) | 18:303 | 5.6 (3.4-8.7) | 418:7620 | 5.2 (4.7-5.7) |
| HI | *GLUD1* | c.1493C>T, p.(Ser498Leu) | 44:870 | 4.8 (3.5-6.4) | 237:4471 | 5.0 (4.4-5.7) |
| HI | *HK1* | Chr10:g.69348891C>T | 29:630 | 4.4 (3.0-6.3) | 166:3261 | 4.8 (4.1-5.6) |
| HI | *HK1* | Chr10:g.69348932_69348935del^‡^ | 21:486 | 4.1 (2.6-6.3) | 204:4999 | 3.9 (3.4-4.5) |
| HI | *GLUD1* | c.820C>T, p.(Arg274Cys) | 22:514 | 4.1 (2.6-6.1) | 269:5011 | 5.1 (4.5-5.7) |
| HI | *GLUD1* | c.953G>A, p.(Arg318Lys) | 21:494 | 4.1 (2.5-6.2) | 317:6277 | 4.8 (4.3-5.4) |
| HI | *GLUD1* | c.1466C>T, p.(Pro489Leu) | 15:386^†^ | 3.7 (2.1-6.1) | 343:9759 | 3.4 (3.1-3.8) |
| HI | *GLUD1* | c.1493C>T, p.(Ser498Leu) | 9:252^†^ | 3.4 (1.6-6.4) | 783:14632 | 5.1 (4.7-5.4) |
| HI | *GCK* | c.1361_1363dup, p.(Ala454dup) | 9:287 | 3.0 (1.4-5.6) | 545:12636 | 4.1 (3.8-4.5) |
| HI | *HK1* | Chr10:g.69348932_69348935del | 13:432 | 2.9 (1.6-4.9) | 309:9588 | 3.1 (2.8-3.5) |
| HI | *GLUD1* | c.1519C>T, p.(His507Tyr) | 18:693 | 2.5 (1.5-4.0) | 177:5533 | 3.1 (2.7-3.6) |
| HI | *GLUD1* | c.1498G>A, p.(Ala500Thr) | 18:724 | 2.4 (1.4-3.8) | 157:6899 | 2.2 (1.9-2.6) |
| HI | *GCK* | c.1361_1363dup, p.(Ala454dup) | 9:512 | 1.7 (0.8-3.3) | 187:9965 | 1.8 (1.6-2.1) |
| HI | *GLUD1* | c.943C>T, p.(His315Tyr) | 12:795^†^ | 1.5 (0.8-2.6) | 88:17944 | 0.5 (0.4-0.6)* |
| HI | *GLUD1* | c.965G>A, p.(Arg322His) | 20:1517^†^ | 1.3 (0.8-2.0) | 52:6759 | 0.8 (0.6-1.0)* |
| HI | *GLUD1* | c.1493C>T, p.(Ser498Leu) | 4:306^†^ | 1.3 (0.4-3.3) | 344:15474 | 2.2 (2.0-2.4) |
| HI | *GCK* | c.1340G>T, p.(Arg447Leu) | 7:599^†^ | 1.2 (0.5-2.4) | 15:2626** | 0.6 (0.3-0.9) |
| HI | *ABCC8* | c.2143G>A, p.(Val715Met)^‡^ | 4:343 | 1.2 (0.3-2.9) | 1:4842 | 0.0 (0.0-0.1) |
| HI | *GLUD1* | c.1493C>T, p.(Ser498Leu) | 4:356^†^ | 1.1 (0.3-2.8) | 182:13198 | 1.4 (1.2-1.6) |
| HI | *ABCC8* | c.4433G>T, p.(Gly1478Val) | 7:628^†§^ | 1.1 (0.4-2.3) | 0:11232 | 0.0 (0.0-0.0) |
| HI | *GLUD1* | c.965G>A, p.(Arg322His) | 8:745 | 1.1 (0.5-2.1) | 46:9614 | 0.5 (0.3-0.6)* |
| HI | *HK1* | Chr10:g.69348891C>T | 6:593^†^ | 1.0 (0.4-2.2) | 40:5936 | 0.7 (0.5-0.9) |
| HI | *ABCC8* | c.1433C>A, p.(Ala478Asp) | 6:676^†§^ | 0.9 (0.3-1.9) | 0:31334 | 0.0 (0.0-0.0) |
| HI | *ABCC8* | c.2143G>A, p.(Val715Met) | 4:464 | 0.9 (0.2-2.2) | 0:8749 | 0.0 (0.0-0.0) |
| HI | *ABCC8* | c.1432G>A, p.(Ala478Thr) | 4:531^†^ | 0.7 (0.2-1.9) | 0:10653 | 0.0 (0.0-0.0) |
| HI | *ABCC8* | c.4151G>A, p.(Gly1384Glu) | 4:554^†^ | 0.7 (0.2-1.8) | 1:9337 | 1. (0.0-0.1) |
| HI | *ABCC8* | c.4435G>A, p.(Gly1479Arg) | 4:565^†^ | 0.7 (0.2-1.8) | 1:9831 | 0.0 (0.0-0.1) |
| HI | *GCK* | c.194C>T, p.(Thr65Ile) | 3:525^†^ | 0.6 (0.1-1.7) | 1:9500 | 0.0 (0.0-0.1) |
| HI | *ABCC8* | c.2147G>A, p.(Gly716Asp) | 6:607^†^ | 0.5 (0.1-1.4) | 1:7382 | 0.0 (0.0-0.1) |
| **ddPCR not possible** | | | | | | |
| HI | *GLUD1* | c.943C>T, p.(His315Tyr) | 8:688^†^ | 1.2 (0.5-2.3) | Not performed, insufficient DNA | |
| HI | *GLUD1* | c.1519C>T, p.(His507Tyr) | 9:827^†^ | 1.1 (0.5-2.0) | Not performed, insufficient DNA | |
| HI | *GCK* | c.205T>C, p.(Ser69Pro) | 4:510^†^ | 0.8 (0.2-2.0) | Not performed, assay failed | |
| HI | *ABCC8* | c.4519G>A, p.(Glu1507Lys) | 3:410^†^ | - 1. (0.2-2.1 | Not performed, insufficient DNA | |
| HI | *ABCC8* | c.4463A>G, p.(Gly1488Arg) | 3:432^†^ | 0.7 (0.1-2.0) | Not performed, insufficient DNA | |


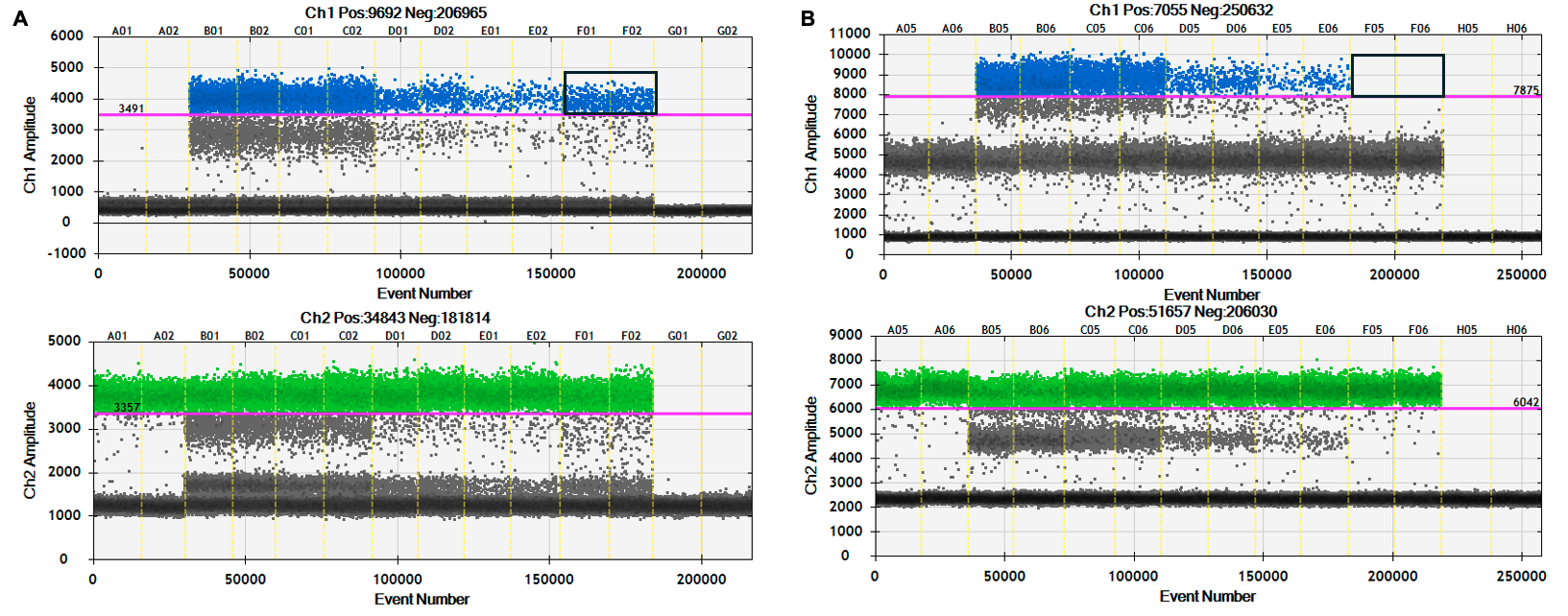


**Supplementary Figure 1**: Example droplet digital PCR (ddPCR) results. Each sample was tested in duplicate. Left to right: normal control without variant of interest (columns A), sample from case heterozygous for variant of interest (B), dilution of heterozygous control to create 25% control (C), 5% control (D), 2% control (E), case to test for the variant of interest (F), and NTC (water) (G). The blue droplets indicate the alternate allele and the green the reference allele. The purple line is the threshold, set at a level in which the 50%, 25%, 5% and 2% controls were close to those values, excluding the ddPCR ‘rain’. A) Example of a positive ddPCR result. Black box indicates droplets positive for alternate allele in the patient sample. B) Example of a negative ddPCR result. Black box indicates lack of droplets positive for alternate allele in the patient sample.

**Supplementary Figure 2**: Scatter plot of targeted next generation sequencing variant allele frequency (VAF) verses droplet digital PCR VAF. Error bars indicate 95% confidence intervals. Black: true positive variants confirmed by droplet digital PCR (ddPCR). Red: false positive variants not confirmed by ddPCR. R-squared value 0.82. Variation in concordance between tNGS and ddPCR can be explained by technical reasons such as sequence context impacting tNGS variant calls e.g. low ddPCR VAF compared to tNGS VAF in some samples due to binding of reference allele probe to alternate allele probe. Blue line represents y=x which would be expected if the relationship between each VAF estimate was 1:1.


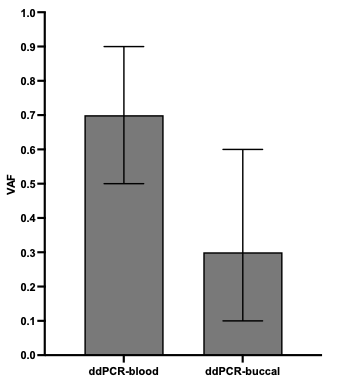


**Supplementary Figure 3:** ddPCR variant allele frequency (VAF) detected from blood and buccal DNA from one individual with a low-level mosaic *HK1* variant (Chr10:g.69348891C>T) Error bars indicate 95% confidence intervals.

**References**

1. Macmullen CM, Zhou Q, Snider KE, Tewson PH, Becker SA, Aziz AR, et al. Diazoxide-unresponsive congenital hyperinsulinism in children with dominant mutations of the beta-cell sulfonylurea receptor SUR1. Diabetes. 2011;60(6):1797-804.

2. Boodhansingh KE, Kandasamy B, Mitteer L, Givler S, De Leon DD, Shyng SL, et al. Novel dominant K(ATP) channel mutations in infants with congenital hyperinsulinism: Validation by in vitro expression studies and in vivo carrier phenotyping. Am J Med Genet A. 2019;179(11):2214-27.

3. Bellanne-Chantelot C, Saint-Martin C, Ribeiro MJ, Vaury C, Verkarre V, Arnoux JB, et al. ABCC8 and KCNJ11 molecular spectrum of 109 patients with diazoxide-unresponsive congenital hyperinsulinism. J Med Genet. 2010;47(11):752-9.

4. De Franco E, Saint-Martin C, Brusgaard K, Knight Johnson AE, Aguilar-Bryan L, Bowman P, et al. Update of variants identified in the pancreatic beta-cell KATP channel genes KCNJ11 and ABCC8 in individuals with congenital hyperinsulinism and diabetes. Hum Mutat. 2020;41(5):884-905.

5. Arya VB, Guemes M, Nessa A, Alam S, Shah P, Gilbert C, et al. Clinical and histological heterogeneity of congenital hyperinsulinism due to paternally inherited heterozygous ABCC8:KCNJ11 mutations. Eur J Endocrinol. 2014;171(6):685-95.

6. Khoriati D, Arya VB, Flanagan SE, Ellard S, Hussain K. Prematurity, macrosomia, hyperinsulinaemic hypoglycaemia and a dominant ABCC8 gene mutation. BMJ Case Rep. 2013;2013.

7. Pinney SE, Ganapathy K, Bradfield J, Stokes D, Sasson A, Mackiewicz K, et al. Dominant form of congenital hyperinsulinism maps to HK1 region on 10q. Horm Res Paediatr. 2013;80(1):18-27.

8. Kapoor RR, Flanagan SE, James CT, McKiernan J, Thomas AM, Harmer SC, et al. Hyperinsulinaemic hypoglycaemia and diabetes mellitus due to dominant ABCC8:KCNJ11 mutations. Diabetologia. 2011;54(10):2575-83.

9. Kapoor RR, Flanagan SE, Arya VB, Shield JP, Ellard S, Hussain K. Clinical and molecular characterisation of 300 patients with congenital hyperinsulinism. Eur J Endocrinol. 2013;168(4):557-64.

10. Flanagan SE, Kapoor RR, Banerjee I, Hall C, Smith VV, Hussain K, et al. Dominantly acting ABCC8 mutations in patients with medically unresponsive hyperinsulinaemic hypoglycaemia. Clin Genet. 2011;79(6):582-7.

11. Mohnike K, Wieland I, Barthlen W, Vogelgesang S, Empting S, Mohnike W, et al. Clinical and genetic evaluation of patients with KATP channel mutations from the German registry for congenital hyperinsulinism. Horm Res Paediatr. 2014;81(3):156-68.

12. Sang Y, Xu Z, Liu M, Yan J, Wu Y, Zhu C, et al. Mutational analysis of ABCC8, KCNJ11, GLUD1, HNF4A and GCK genes in 30 Chinese patients with congenital hyperinsulinism. Endocr J. 2014;61(9):901-10.

13. Huopio H, Reimann F, Ashfield R, Komulainen J, Lenko HL, Rahier J, et al. Dominantly inherited hyperinsulinism caused by a mutation in the sulfonylurea receptor type 1. J Clin Invest. 2000;106(7):897-906.

14. Salomon-Estebanez M, Flanagan SE, Ellard S, Rigby L, Bowden L, Mohamed Z, et al. Conservatively treated Congenital Hyperinsulinism (CHI) due to K-ATP channel gene mutations: reducing severity over time. Orphanet J Rare Dis. 2016;11(1):163.

15. Christesen HB, Tribble ND, Molven A, Siddiqui J, Sandal T, Brusgaard K, et al. Activating glucokinase (GCK) mutations as a cause of medically responsive congenital hyperinsulinism: prevalence in children and characterisation of a novel GCK mutation. Eur J Endocrinol. 2008;159(1):27-34.

16. Mannisto JME, Maria M, Raivo J, Kuulasmaa T, Otonkoski T, Huopio H, et al. Clinical and Genetic Characterization of 153 Patients with Persistent or Transient Congenital Hyperinsulinism. J Clin Endocrinol Metab. 2020;105(4).

17. Wabitsch M, Lahr G, Van de Bunt M, Marchant C, Lindner M, von Puttkamer J, et al. Heterogeneity in disease severity in a family with a novel G68V GCK activating mutation causing persistent hyperinsulinaemic hypoglycaemia of infancy. Diabet Med. 2007;24(12):1393-9.

18. Li C, Juliana CA, Yuan Y, Li M, Lu M, Chen P, et al. Phenotypic Characterization of Congenital Hyperinsulinism Due to Novel Activating Glucokinase Mutations. Diabetes. 2023;72(12):1809-19.

19. Koneshamoorthy A, Seniveratne-Epa D, Calder G, Sawyer M, Kay TWH, Farrell S, et al. Case Report: Hypoglycemia Due to a Novel Activating Glucokinase Variant in an Adult - a Molecular Approach. Front Endocrinol (Lausanne). 2022;13:842937.

20. Beer NL, van de Bunt M, Colclough K, Lukacs C, Arundel P, Chik CL, et al. Discovery of a novel site regulating glucokinase activity following characterization of a new mutation causing hyperinsulinemic hypoglycemia in humans. J Biol Chem. 2011;286(21):19118-26.

21. Sayed S, Langdon DR, Odili S, Chen P, Buettger C, Schiffman AB, et al. Extremes of clinical and enzymatic phenotypes in children with hyperinsulinism caused by glucokinase activating mutations. Diabetes. 2009;58(6):1419-27.

22. Cuesta-Munoz AL, Huopio H, Otonkoski T, Gomez-Zumaquero JM, Nanto-Salonen K, Rahier J, et al. Severe persistent hyperinsulinemic hypoglycemia due to a de novo glucokinase mutation. Diabetes. 2004;53(8):2164-8.

23. Boodhansingh KE, Yang Z, Li C, Chen P, Lord K, Becker SA, et al. Localized islet nuclear enlargement hyperinsulinism (LINE-HI) due to ABCC8 and GCK mosaic mutations. Eur J Endocrinol. 2022;187(2):301-13.

24. Kapoor RR, Flanagan SE, Fulton P, Chakrapani A, Chadefaux B, Ben-Omran T, et al. Hyperinsulinism-hyperammonaemia syndrome: novel mutations in the GLUD1 gene and genotype-phenotype correlations. Eur J Endocrinol. 2009;161(5):731-5.

25. Roy K, Satapathy AK, Houhton JAL, Flanagan SE, Radha V, Mohan V, et al. Congenital Hyperinsulinemic Hypoglycemia and Hyperammonemia due to Pathogenic Variants in GLUD1. Indian J Pediatr. 2019;86(11):1051-3.

26. Stanley CA, Fang J, Kutyna K, Hsu BY, Ming JE, Glaser B, et al. Molecular basis and characterization of the hyperinsulinism:hyperammonemia syndrome: predominance of mutations in exons 11 and 12 of the glutamate dehydrogenase gene. HI:HA Contributing Investigators. Diabetes. 2000;49(4):667-73.

27. Stanley CA, Lieu YK, Hsu BY, Burlina AB, Greenberg CR, Hopwood NJ, et al. Hyperinsulinism and hyperammonemia in infants with regulatory mutations of the glutamate dehydrogenase gene. N Engl J Med. 1998;338(19):1352-7.

28. Bahi-Buisson N, Roze E, Dionisi C, Escande F, Valayannopoulos V, Feillet F, et al. Neurological aspects of hyperinsulinism-hyperammonaemia syndrome. Dev Med Child Neurol. 2008;50(12):945-9.

29. Aftab S, Gubaeva D, Houghton JAL, Dastamani A, Sotiridou E, Gilbert C, et al. Spectrum of neuro-developmental disorders in children with congenital hyperinsulinism due to activating mutations in GLUD1. Endocr Connect. 2023;12(4).

30. Bennett JJ, Saint-Martin C, Neumann B, Mannisto JME, Houghton JAL, Empting S, et al. Non-coding cis-regulatory variants in HK1 cause congenital hyperinsulinism with variable disease severity. Genome Med. 2025;17(1):17.

31. Wakeling MN, Owens NDL, Hopkinson JR, Johnson MB, Houghton JAL, Dastamani A, et al. Non-coding variants disrupting a tissue-specific regulatory element in HK1 cause congenital hyperinsulinism. Nat Genet. 2022;54(11):1615-20.

32. Zwaveling-Soonawala N, Hagebeuk EE, Slingerland AS, Ris-Stalpers C, Vulsma T, van Trotsenburg AS. Successful transfer to sulfonylurea therapy in an infant with developmental delay, epilepsy and neonatal diabetes (DEND) syndrome and a novel ABCC8 gene mutation. Diabetologia. 2011;54(2):469-71.

33. Jahnavi S, Poovazhagi V, Mohan V, Bodhini D, Raghupathy P, Amutha A, et al. Clinical and molecular characterization of neonatal diabetes and monogenic syndromic diabetes in Asian Indian children. Clin Genet. 2013;83(5):439-45.

34. Proks P, Arnold AL, Bruining J, Girard C, Flanagan SE, Larkin B, et al. A heterozygous activating mutation in the sulphonylurea receptor SUR1 (ABCC8) causes neonatal diabetes. Hum Mol Genet. 2006;15(11):1793-800.

35. Ellard S, Flanagan SE, Girard CA, Patch AM, Harries LW, Parrish A, et al. Permanent neonatal diabetes caused by dominant, recessive, or compound heterozygous SUR1 mutations with opposite functional effects. Am J Hum Genet. 2007;81(2):375-82.

36. Patch AM, Flanagan SE, Boustred C, Hattersley AT, Ellard S. Mutations in the ABCC8 gene encoding the SUR1 subunit of the KATP channel cause transient neonatal diabetes, permanent neonatal diabetes or permanent diabetes diagnosed outside the neonatal period. Diabetes Obes Metab. 2007;9 Suppl 2(Suppl 2):28-39.

37. Rafiq M, Flanagan SE, Patch AM, Shields BM, Ellard S, Hattersley AT, et al. Effective treatment with oral sulfonylureas in patients with diabetes due to sulfonylurea receptor 1 (SUR1) mutations. Diabetes Care. 2008;31(2):204-9.

38. Flanagan SE, Patch AM, Mackay DJ, Edghill EL, Gloyn AL, Robinson D, et al. Mutations in ATP-sensitive K+ channel genes cause transient neonatal diabetes and permanent diabetes in childhood or adulthood. Diabetes. 2007;56(7):1930-7.

39. Codner E, Flanagan SE, Ugarte F, Garcia H, Vidal T, Ellard S, et al. Sulfonylurea treatment in young children with neonatal diabetes: dealing with hyperglycemia, hypoglycemia, and sick days. Diabetes Care. 2007;30(5):e28-9.

40. Rubio-Cabezas O, Flanagan SE, Damhuis A, Hattersley AT, Ellard S. KATP channel mutations in infants with permanent diabetes diagnosed after 6 months of life. Pediatr Diabetes. 2012;13(4):322-5.

41. Demirbilek H, Arya VB, Ozbek MN, Houghton JA, Baran RT, Akar M, et al. Clinical characteristics and molecular genetic analysis of 22 patients with neonatal diabetes from the South-Eastern region of Turkey: predominance of non-KATP channel mutations. Eur J Endocrinol. 2015;172(6):697-705.

42. Globa E, Zelinska N, Mackay DJ, Temple KI, Houghton JA, Hattersley AT, et al. Neonatal diabetes in Ukraine: incidence, genetics, clinical phenotype and treatment. J Pediatr Endocrinol Metab. 2015;28(11-12):1279-86.

43. Zhang M, Chen X, Shen S, Li T, Chen L, Hu M, et al. Sulfonylurea in the treatment of neonatal diabetes mellitus children with heterogeneous genetic backgrounds. J Pediatr Endocrinol Metab. 2015;28(7-8):877-84.

44. Klee P, Bellanne-Chantelot C, Depret G, Llano JP, Paget C, Nicolino M. A novel ABCC8 mutation illustrates the variability of the diabetes phenotypes associated with a single mutation. Diabetes Metab. 2012;38(2):179-82.

45. Babenko AP, Polak M, Cave H, Busiah K, Czernichow P, Scharfmann R, et al. Activating mutations in the ABCC8 gene in neonatal diabetes mellitus. N Engl J Med. 2006;355(5):456-66.

46. Suzuki S, Makita Y, Mukai T, Matsuo K, Ueda O, Fujieda K. Molecular basis of neonatal diabetes in Japanese patients. J Clin Endocrinol Metab. 2007;92(10):3979-85.

47. Hashimoto Y, Dateki S, Hirose M, Satomura K, Sawada H, Mizuno H, et al. Molecular and clinical features of K(ATP) -channel neonatal diabetes mellitus in Japan. Pediatr Diabetes. 2017;18(7):532-9.

48. Jain V, Flanagan SE, Ellard S. Permanent neonatal diabetes caused by a novel mutation. Indian Pediatr. 2012;49(6):486-8.

49. Artuso R, Provenzano A, Mazzinghi B, Giunti L, Palazzo V, Andreucci E, et al. Therapeutic implications of novel mutations of the RFX6 gene associated with early-onset diabetes. Pharmacogenomics J. 2015;15(1):49-54.

50. Mannikko R, Flanagan SE, Sim X, Segal D, Hussain K, Ellard S, et al. Mutations of the same conserved glutamate residue in NBD2 of the sulfonylurea receptor 1 subunit of the KATP channel can result in either hyperinsulinism or neonatal diabetes. Diabetes. 2011;60(6):1813-22.

51. Iafusco D, Massa O, Pasquino B, Colombo C, Iughetti L, Bizzarri C, et al. Minimal incidence of neonatal:infancy onset diabetes in Italy is 1:90,000 live births. Acta Diabetol. 2012;49(5):405-8.

52. Vaxillaire M, Dechaume A, Busiah K, Cave H, Pereira S, Scharfmann R, et al. New ABCC8 mutations in relapsing neonatal diabetes and clinical features. Diabetes. 2007;56(6):1737-41.

53. Taberner P, Flanagan SE, Mackay DJ, Ellard S, Taverna MJ, Ferraro M. Clinical and genetic features of Argentinian children with diabetes-onset before 12months of age: Successful transfer from insulin to oral sulfonylurea. Diabetes Res Clin Pract. 2016;117:104-10.

54. Garin I, Perez de Nanclares G, Gastaldo E, Harries LW, Rubio-Cabezas O, Castano L. Permanent neonatal diabetes caused by creation of an ectopic splice site within the INS gene. PLoS One. 2012;7(1):e29205.

55. Stoy J, Edghill EL, Flanagan SE, Ye H, Paz VP, Pluzhnikov A, et al. Insulin gene mutations as a cause of permanent neonatal diabetes. Proc Natl Acad Sci U S A. 2007;104(38):15040-4.

56. Dimova R, Tankova T, Gergelcheva I, Tournev I, Konstantinova M. A family with permanent neonatal diabetes due to a novel mutation in INS gene. Diabetes Res Clin Pract. 2015;108(2):e28-30.

57. Catli G, Abaci A, Flanagan SE, Anik A, Ellard S, Bober E. Permanent neonatal diabetes caused by a novel mutation in the INS gene. Diabetes Res Clin Pract. 2013;99(1):e5-8.

58. Ngoc CTB, Dung VC, De Franco E, Lan NN, Thao BP, Khanh NN, et al. Genetic Etiology of Neonatal Diabetes Mellitus in Vietnamese Infants and Characteristics of Those With INS Gene Mutations. Front Endocrinol (Lausanne). 2022;13:866573.

59. Edghill EL, Flanagan SE, Patch AM, Boustred C, Parrish A, Shields B, et al. Insulin mutation screening in 1,044 patients with diabetes: mutations in the INS gene are a common cause of neonatal diabetes but a rare cause of diabetes diagnosed in childhood or adulthood. Diabetes. 2008;57(4):1034-42.

60. Craig TJ, Shimomura K, Holl RW, Flanagan SE, Ellard S, Ashcroft FM. An in-frame deletion in Kir6.2 (KCNJ11) causing neonatal diabetes reveals a site of interaction between Kir6.2 and SUR1. J Clin Endocrinol Metab. 2009;94(7):2551-7.

61. Flanagan SE, Edghill EL, Gloyn AL, Ellard S, Hattersley AT. Mutations in KCNJ11, which encodes Kir6.2, are a common cause of diabetes diagnosed in the first 6 months of life, with the phenotype determined by genotype. Diabetologia. 2006;49(6):1190-7.

62. Mlynarski W, Tarasov AI, Gach A, Girard CA, Pietrzak I, Zubcevic L, et al. Sulfonylurea improves CNS function in a case of intermediate DEND syndrome caused by a mutation in KCNJ11. Nat Clin Pract Neurol. 2007;3(11):640-5.

63. Shahawy S, Chan NK, Ellard S, Young E, Shahawy H, Mace J, et al. A pathway to insulin independence in newborns and infants with diabetes. J Perinatol. 2011;31(8):567-70.

64. Massa O, Iafusco D, D'Amato E, Gloyn AL, Hattersley AT, Pasquino B, et al. KCNJ11 activating mutations in Italian patients with permanent neonatal diabetes. Hum Mutat. 2005;25(1):22-7.

65. Flanagan SE, Patch AM, Ellard S. Using SIFT and PolyPhen to predict loss-of-function and gain-of-function mutations. Genet Test Mol Biomarkers. 2010;14(4):533-7.

66. Gloyn AL, Pearson ER, Antcliff JF, Proks P, Bruining GJ, Slingerland AS, et al. Activating mutations in the gene encoding the ATP-sensitive potassium-channel subunit Kir6.2 and permanent neonatal diabetes. N Engl J Med. 2004;350(18):1838-49.

67. Doneray H, Houghton J, Tekgunduz KS, Balkir F, Caner I. Permanent neonatal diabetes mellitus caused by a novel mutation in the KCNJ11 gene. J Pediatr Endocrinol Metab. 2014;27(3-4):367-71.

68. Gloyn AL, Reimann F, Girard C, Edghill EL, Proks P, Pearson ER, et al. Relapsing diabetes can result from moderately activating mutations in KCNJ11. Hum Mol Genet. 2005;14(7):925-34.

69. Khadilkar VV, Khadilkar AV, Kapoor RR, Hussain K, Hattersley AT, Ellard S. KCNJ11 activating mutation in an Indian family with remitting and relapsing diabetes. Indian J Pediatr. 2010;77(5):551-4.

70. Mannikko R, Jefferies C, Flanagan SE, Hattersley A, Ellard S, Ashcroft FM. Interaction between mutations in the slide helix of Kir6.2 associated with neonatal diabetes and neurological symptoms. Hum Mol Genet. 2010;19(6):963-72.

71. Mannikko R, Stansfeld PJ, Ashcroft AS, Hattersley AT, Sansom MS, Ellard S, et al. A conserved tryptophan at the membrane-water interface acts as a gatekeeper for Kir6.2:SUR1 channels and causes neonatal diabetes when mutated. J Physiol. 2011;589(Pt 13):3071-83.

72. O'Connell SM, Proks P, Kramer H, Mattis KK, Sachse G, Joyce C, et al. The value of in vitro studies in a case of neonatal diabetes with a novel Kir6.2-W68G mutation. Clin Case Rep. 2015;3(10):884-7.

73. Babiker T, Vedovato N, Patel K, Thomas N, Finn R, Mannikko R, et al. Successful transfer to sulfonylureas in KCNJ11 neonatal diabetes is determined by the mutation and duration of diabetes. Diabetologia. 2016;59(6):1162-6.

74. Gloyn AL, Diatloff-Zito C, Edghill EL, Bellanne-Chantelot C, Nivot S, Coutant R, et al. KCNJ11 activating mutations are associated with developmental delay, epilepsy and neonatal diabetes syndrome and other neurological features. Eur J Hum Genet. 2006;14(7):824-30.

75. Shimomura K, Horster F, de Wet H, Flanagan SE, Ellard S, Hattersley AT, et al. A novel mutation causing DEND syndrome: a treatable channelopathy of pancreas and brain. Neurology. 2007;69(13):1342-9.

76. Bonnefond A, Philippe J, Durand E, Muller J, Saeed S, Arslan M, et al. Highly sensitive diagnosis of 43 monogenic forms of diabetes or obesity through one-step PCR-based enrichment in combination with next-generation sequencing. Diabetes Care. 2014;37(2):460-7.

77. Shimomura K, de Nanclares GP, Foutinou C, Caimari M, Castano L, Ashcroft FM. The first clinical case of a mutation at residue K185 of Kir6.2 (KCNJ11): a major ATP-binding residue. Diabet Med. 2010;27(2):225-9.

78. Russo L, Iafusco D, Brescianini S, Nocerino V, Bizzarri C, Toni S, et al. Permanent diabetes during the first year of life: multiple gene screening in 54 patients. Diabetologia. 2011;54(7):1693-701.

79. Codner E, Flanagan S, Ellard S, Garcia H, Hattersley AT. High-dose glibenclamide can replace insulin therapy despite transitory diarrhea in early-onset diabetes caused by a novel R201L Kir6.2 mutation. Diabetes Care. 2005;28(3):758-9.

80. Edghill EL, Gloyn AL, Goriely A, Harries LW, Flanagan SE, Rankin J, et al. Origin of de novo KCNJ11 mutations and risk of neonatal diabetes for subsequent siblings. J Clin Endocrinol Metab. 2007;92(5):1773-7.

81. Joshi R, Phatarpekar A. Neonatal diabetes mellitus due to L233F mutation in the KCNJ11 gene. World J Pediatr. 2011;7(4):371-2.

82. Jesic MM, Jesic MD, Maglajlic S, Sajic S, Necic S. Successful sulfonylurea treatment of a neonate with neonatal diabetes mellitus due to a new KCNJ11 mutation. Diabetes Res Clin Pract. 2011;91(1):e1-3.

83. Girard CA, Shimomura K, Proks P, Absalom N, Castano L, Perez de Nanclares G, et al. Functional analysis of six Kir6.2 (KCNJ11) mutations causing neonatal diabetes. Pflugers Arch. 2006;453(3):323-32.

84. Gole E, Oikonomou S, Ellard S, De Franco E, Karavanaki K. A Novel KCNJ11 Mutation Associated with Transient Neonatal Diabetes. J Clin Res Pediatr Endocrinol. 2018;10(2):175-8.

85. Busiah K, Verkarre V, Cave H, Scharfmann R, Polak M. Human pancreas endocrine cell populations and activating ABCC8 mutations. Horm Res Paediatr. 2014;82(1):59-64.

86. Shimomura K, Flanagan SE, Zadek B, Lethby M, Zubcevic L, Girard CA, et al. Adjacent mutations in the gating loop of Kir6.2 produce neonatal diabetes and hyperinsulinism. EMBO Mol Med. 2009;1(3):166-77.

87. Tarasov AI, Girard CA, Larkin B, Tammaro P, Flanagan SE, Ellard S, et al. Functional analysis of two Kir6.2 (KCNJ11) mutations, K170T and E322K, causing neonatal diabetes. Diabetes Obes Metab. 2007;9 Suppl 2:46-55.

88. Flechtner I, Vaxillaire M, Cave H, Scharfmann R, Froguel P, Polak M. Neonatal hyperglycaemia and abnormal development of the pancreas. Best Pract Res Clin Endocrinol Metab. 2008;22(1):17-40.

89. Philla KQ, Bauer AJ, Vogt KS, Greeley SA. Successful transition from insulin to sulfonylurea therapy in a patient with monogenic neonatal diabetes owing to a KCNJ11 F333L [corrected] mutation. Diabetes Care. 2013;36(12):e201.

90. Masia R, Koster JC, Tumini S, Chiarelli F, Colombo C, Nichols CG, et al. An ATP-binding mutation (G334D) in KCNJ11 is associated with a sulfonylurea-insensitive form of developmental delay, epilepsy, and neonatal diabetes. Diabetes. 2007;56(2):328-36.

91. Flanagan SE, De Franco E, Lango Allen H, Zerah M, Abdul-Rasoul MM, Edge JA, et al. Analysis of transcription factors key for mouse pancreatic development establishes NKX2-2 and MNX1 mutations as causes of neonatal diabetes in man. Cell Metab. 2014;19(1):146-54.
